# Epstein-Barr Virus Enhances Cancer-Specific Aberrant Splicing of TSG101 Pre-mRNA

**DOI:** 10.3390/ijms23052516

**Published:** 2022-02-24

**Authors:** Huey-Huey Chua, Toshiki Kameyama, Akila Mayeda, Te-Huei Yeh

**Affiliations:** 1 Department of Pediatrics, National Taiwan University Hospital, College of Medicine, National Taiwan University, Taipei 100226, Taiwan; hueyhueychua@gmail.com; 2 Department of Physiology, School of Medicine, Fujita Health University, Toyoake 470-1192, Aichi, Japan; tkame@fujita-hu.ac.jp; 3 Division of Gene Expression Mechanism, Institute for Comprehensive Medical Science, Fujita Health University, Toyoake 470-1192, Aichi, Japan; 4 Department of Otolaryngology, National Taiwan University Hospital, College of Medicine, National Taiwan University, Taipei 100225, Taiwan

**Keywords:** TSG101, Epstein-Barr virus, lytic cycle, EBNA-1, Zta, Rta, VCA, gp350/220, nasopharyngeal carcinoma, Burkitt lymphoma

## Abstract

Tumor viruses gain control of cellular functions when they infect and transform host cells. Alternative splicing is one of the cellular processes exploited by tumor viruses to benefit viral replication and support oncogenesis. Epstein-Barr virus (EBV) participates in a number of cancers, as reported mostly in nasopharyngeal carcinoma (NPC) and Burkitt lymphoma (BL). Using RT-nested-PCR and Northern blot analysis in NPC and BL cells, here we demonstrate that EBV promotes specific alternative splicing of TSG101 pre-mRNA, which generates the TSG101∆154-1054 variant though the agency of its viral proteins, such as EBNA-1, Zta and Rta. The level of TSG101∆154-1054 is particularly enhanced upon EBV entry into the lytic cycle, increasing protein stability of TSG101 and causing the cumulative synthesis of EBV late lytic proteins, such as VCA and gp350/220. TSG101∆154-1054-mediated production of VCA and gp350/220 is blocked by the overexpression of a translational mutant of TSG101∆154-1054 or by the depletion of full-length TSG101, which is consistent with the known role of the TSG101∆154-1054 protein in stabilizing the TSG101 protein. NPC patients whose tumor tissues express TSG101∆154-1054 have high serum levels of anti-VCA antibodies and high levels of viral DNA in their tumors. Our findings highlight the functional importance of TSG101∆154-1054 in allowing full completion of the EBV lytic cycle to produce viral particles. We propose that targeting EBV-induced TSG101 alternative splicing has broad potential as a therapeutic to treat EBV-associated malignancies.

## 1. Introduction

Epstein-Barr virus (EBV) is an enveloped γ-herpesvirus carrying a double-stranded DNA genome that is a well-recognized tumor virus [1]. It can immortalize primary B cells and certain epithelial cells [2]. Burkitt’s lymphoma (BL), nasopharyngeal carcinoma (NPC), post-transplant B cell lymphomas, Hodgkin’s disease, and gastric carcinoma [2,3] are the typical EBV-associated malignancies, as evidenced by the frequent presence of EBV gene products in tumor biopsies and a strong correlation between EBV viral load in patients’ sera and disease stages [2,3]. After infection, EBV favorably maintains latency by expressing latent proteins such as EBNA-1 (EBV nuclear antigen-1), while it periodically enters the replicative lytic cycle to generate infectious viral particles for transmission [1]. Its immediate early lytic proteins, Zta and Rta, are key transactivators for the switch to the lytic cycle. They turn on the expression of early lytic genes, including *DNA polymerase* and *Early antigen-diffuse (EA-D)*, for viral DNA replication. They then sequentially activate the late lytic genes, e.g., *viral capsid antigen (VCA)* and *gp350/220*, which encode structural proteins to form viral capsids and envelopes [1].

To meet the demand of viral propagation, tumor viruses exploit the cellular resources. As seen in EBV, Zta and Rta redirect the cell-cycle progression to arrest at the Go/G1 and G1/S boundaries, respectively [4,5], so that the resources are diverted to accommodate the replicative program of viral, but not the cellular, DNA. On the other hand, tumor viruses including EBV are capable of reprogramming the host cell’s splicing machinery to generate splicing variants that promote viral gene transcription to evade an immune response, supporting cell proliferation and carcinogenesis [6].

Here we aimed to explore whether EBV impacts on the alternative splicing of TSG101 (tumor susceptibility gene 101), a frequent target of cancer-specific splicing. TSG101 is potentially an oncogene with multiple cellular functions; TSG101 is crucial for cell survival, proliferation, cell-cycle progression, migration and chemoresistance. It is also a transcriptional co-regulator and protein sorter that regulates multiple cellular functions [7,8,9,10,11,12]. A variety of aberrant messages are generated upon the alternative splicing of TSG101 in tumor cells. For example, a major isoform with nucleotides 154 to 1054 spliced out, designated TSG101∆154-1054, is typically found in proliferative cell lines, tumor and fetal tissues but rarely in adult normal cells [13,14,15,16,17,18]. Increased frequency of TSG101∆154-1054 (hereafter referred to as TSG∆154-1054) is observed in advanced neoplastic lesions and during the development of metastasis [19,20]. Remarkably, TSG∆154-1054 is produced via a re-splicing of the ‘normally’ spliced TSG101 mRNA, in a *TSG101* mutation-independent manner [21]. In healthy normal cells, the re-splicing of TSG101 to generate TSG∆154-1054 is repressed by core exon junction complexes comprised of EIF4A3 (eukaryotic translation initiation factor 4A3), MAGOH (mago homolog) and RBM8A (RNA binding motif protein 8A, also known as Y14) [22]. Although the underlying mechanisms remain to be elucidated, the generation of TSG∆154-1054 in cancer cells is tightly correlated with ageing, high histological tumor grade, stresses such as hypoxia and X-ray, and dysfunction and inactivation of p53 [18,23,24,25,26].

Loss of fidelity in the splicing process is frequent during tumorigenesis. We hypothesized that the infection of tumor viruses like EBV is implicated in the dysfunction of alternative splicing machinery for strengthening the oncogenicity of tumor cells, while also promoting efficient viral growth. Here we delineate how EBV regulates the expression of TSG101 via virally induced alternative splicing, which sheds light on the completion mechanism of the productive viral life cycle.

## 2. Results

### 2.1. EBV Triggers Cancer-Specific Alternative Splicing of TSG101

**Figure 1:**
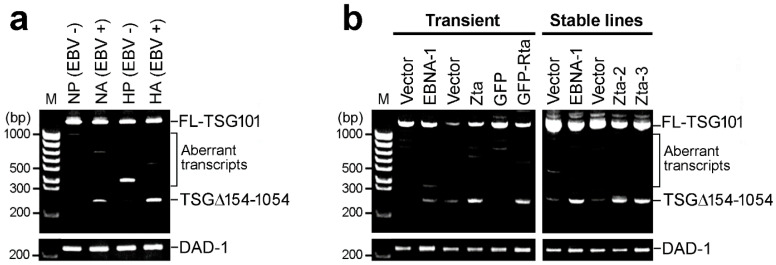
EBV infection triggers the alternative splicing of TSG101 via the viral-encoded EBNA-1, Zta and Rta. (**a**) TSG101-specific RT-nested-PCR was performed on RNA isolated from pZip-transfected NPC-TW01 and HONE-1 stable cell lines (NP and HP), served as EBV-negative controls, and their paired with the Akata strain EBV-positive stable cell lines (NA and HA). (**b**) NPC-TW01 cells transient transfected with EBNA-1, Zta, GFP-Rta and their paired vector controls were subjected to RT-nested-PCR targeting TSG101. Stable RHEK-1 cell lines expressing EBNA-1 and Zta (Zta-2 and Zta-3) were assayed in parallel.

EBV has been reported to drive cellular splicing via its latent protein EBNA-1 [27]. To develop more evidentiary support for EBV elicited alternative splicing of cellular mRNAs, we performed RT-nested-PCR analysis in the EBV-infected NPC-TW01 and HONE-1 stable lines (NA and HA), as well as paired EBV-negative controls (NP and HP), to identify alternative splicing events of TSG101. Full-length TSG101 mRNA was precisely re-spliced to yield the TSG∆154-1054 variant in the presence of EBV (Figure 1a). Remarkably, transient and stable transfection of EBV viral proteins EBNA-1, Zta and Rta enriched the total pool of TSG∆154-1054 isoform as compared to their paired vector controls (Figure 1b), suggesting that EBV promotes re-splicing to generate TSG∆154-1054 via its viral-encoded latent antigen EBNA-1 and lytic transactivators Zta and Rta.

### 2.2. EBV Preferentially Enhances TSG∆154-1054 Production upon Lytic Activation

**Figure 2:**
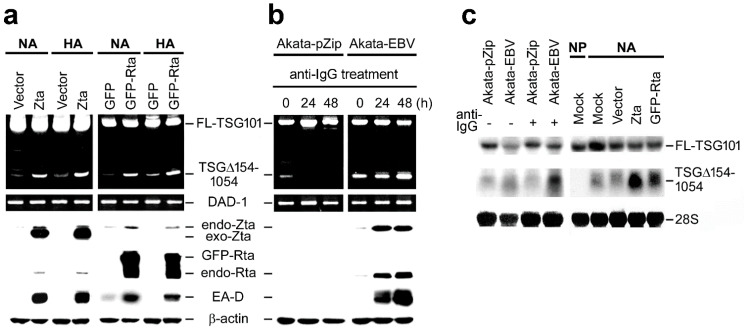
TSG∆154-1054 is specifically induced by the EBV lytic cycle. (**a**,**b**) The level of TSG∆154-1054 was assessed by TSG101-specific RT-nested-PCR (upper) and the progression of the EBV lytic cycle was validated by immunoblotting (lower), using EBV-infected NA and HA cells (**a**), as well as anti-IgG-treated Akata stable cell lines (**b**). The EBV lytic cycle was induced by transient transfection of Zta and GFP-Rta (**a**) or anti-IgG crosslinking (**b**). Expressions of endogenous (endo) and exogenous (exo) transfected Zta and Rta were monitored by immunoblotting. EA-D and β-actin were detected to ensure the progression of the EBV lytic cycle and equal loading of protein lysates, respectively. (**c**) Northern blotting validated the expression levels of TSG∆154-1054 and full-length (FL)-TSG101. 28S rRNA on the same membrane, stained with methylene blue, was used as a loading control.

The expression level of TSG∆154-1054 was compared during EBV latency and lytic reactivation. To induce the EBV lytic cycle, NA and HA cells were transfected with either Zta or GFP-Rta plasmids. Successful entry into the viral lytic cycle was indicated by the expression of EA-D; accordingly, we observed a heightened generation of TSG∆154-1054 generation at the onset of lytic activation (Figure 2a).

Next, we also examined TSG∆154-1054 induction in EBV-positive BL cells, Akata-EBV, and the control EBV-negative cells, Akata-pZip (see Section 4). To induce the EBV lytic cycle, we used an anti-IgG crosslinking assay. The crosslinking of anti-IgG antibodies against surface IgG molecules of Akata-EBV cells induces the PKC-dependent signal transduction cascade, leading to the transcriptional activation of Zta, Rta and EA-D through the expression of early growth response genes (egr1–3) and nuclear orphan receptors (nr4a1, 3) [28]. We observed that both control EBV-negative Akata-pZip and EBV-positive Akata-EBV cell lines remained TSG∆154-1054-positive in standard cultivation conditions (Figure 2b). Upon anti-IgG administration, Akata-pZip lost, but Akata-EBV increased, the level of variant TSG∆154-1054, suggesting that the anti-IgG-induced expression of Zta and Rta stimulated rapid re-splicing of TSG∆154-1054 in Akata-EBV cells (Figure 2b). Northern blot analyses confirmed a further increase of TSG∆154-1054 upon lytic induction in anti-IgG-treated Akata-EBV, as well as Zta- and GFP-Rta-overexpressing NA cells (Figure 2c).

### 2.3. TSG∆154-1054 Increases TSG101 Protein Stability

**Figure 3:**
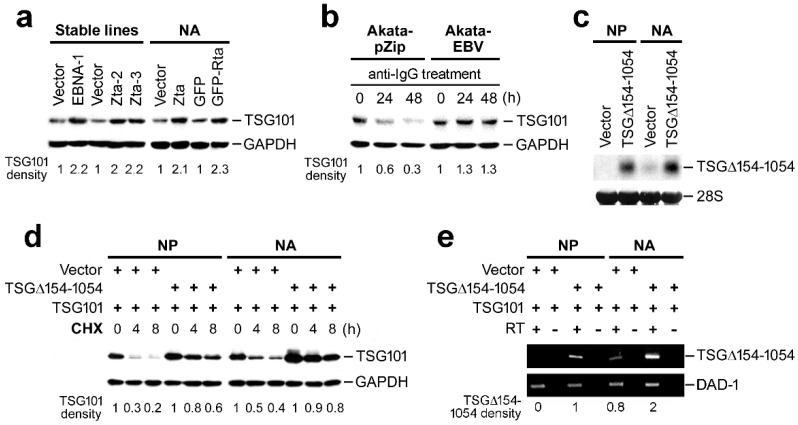
Generation of TSG∆154-1054 is associated with increasing TSG101 stability in an EBV-independent manner. (**a**,**b**) The level of TSG101 protein was analyzed by immunoblotting. We used stable RHEK cell lines expressing the indicated viral proteins and NA cells transfected with the indicated plasmids (**a**), as well as anti-IgG-treated Akata stable cell lines (**b**). Probing of GAPDH was used as a protein loading control for immunoblots. The density of TSG101 protein was normalized to GAPDH and indicated as ratios to the value of control (=1). (**c**) The expression efficiency of TSG∆154-1054 in both NP and NA cells was assessed by Northern blotting. The 28S ribosomal RNA bands indicate the loading amounts of total RNA. (**d**) The degradation of TSG101 was monitored by a CHX-chase assay in NP and NA cells transfected with indicated plasmids. TSG101 protein was detected by immunoblotting and the relative intensity was calculated as described in (**b**). (**e**) The amount of TSG∆154-1054 in transfected cells, at CHX 0 h time point in (**d**), was estimated by RT-PCR. The density of TSG∆154-1054 was normalized to that of DAD-1 and the relative values are indicated.

To examine the effect of TSG∆154-1054, we performed immunoblot analysis in cells expressing TSG∆154-1054 and controls. We observed an enrichment of TSG101 protein in TSG∆154-1054-expressing cells, including EBNA-1 and Zta stable lines, and cells with EBV reactivation, i.e., Zta- and GFP-Rta-introduced NA cells, and anti-IgG-treated Akata-EBV cells (Figure 3a,b; Cf. Figure 2a,b for TSG∆154-1054 expression). Akata-pZip cells failed to preserve TSG∆154-1054 levels during anti-IgG treatment and the total pool of TSG101 was decreased (Figure 3b; Cf. Figure 2b) consistently. These observations are in agreement with our previous findings that TSG∆154-1054 truncated product stabilizes TSG101 wild-type protein [18,26].

However, we do not know whether the EBV-infected cells were capable of a substantial increase in TSG101 stability. To address this issue, we overexpressed TSG∆154-1054 in both EBV-infected NA cells and control NP cells. A Northern blot analysis confirmed nearly equivalent expression levels from the TSG∆154-1054 construct in NP and NA cells (Figure 3c). Thereafter, we estimated the half-life of TSG101 protein in the time-series of a cycloheximide (CHX) chase assay and the result was assessed by immunoblotting. We detected that ectopic expression of TSG∆154-1054 protected TSG101 protein from degradation in both NP and NA cells, and thus the stabilization of TSG101 occurs irrespective of EBV infection (Figure 3d). The difference between vector-transfected NP and NA cells revealed that a low level of endogenous TSG∆154-1054 in NA cells was sufficient to stabilize TSG101 (Figure 3c,d). This was not observed in the vector-transfected NP cells in which the endogenous TSG∆154-1054 was not expressed at all (Figure 3c,d). TSG101, in NA cells, was further stabilized by ectopic overexpression of TSG∆154-1054 (Figure 3d). The extent of the TSG101 stabilization in NP and NA cells paralleled the expression level of TSG∆154-1054 as measured by RT-PCR. DAD-1 (defender against apoptotic death) was included as an internal control (Figure 3d,e).

### 2.4. EBV Exploits TSG101 Stabilization for Viral Late Protein Synthesis

**Figure 4:**
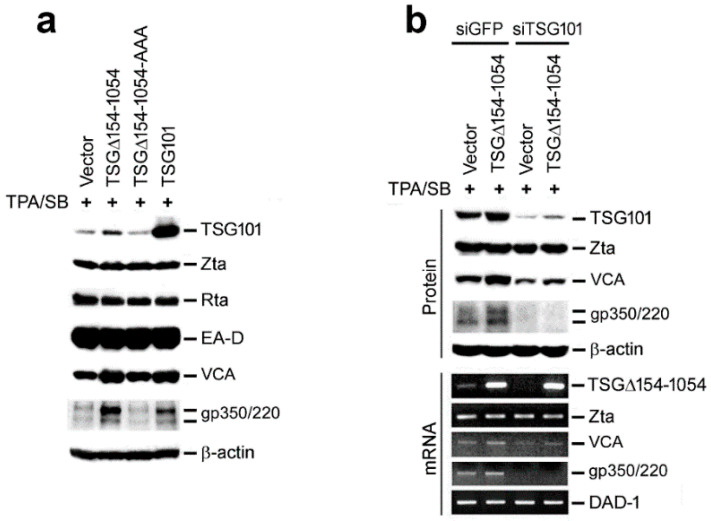
TSG∆154-1054 enhances the expression of EBV late lytic proteins by stabilizing TSG101. (**a**) TSG101 and EBV lytic proteins were detected by immunoblotting using NA cells that were transfected with plasmids encoding TSG∆154-1054, translational mutant TSG∆154-1054-AAA and wild-type TSG101. TPA/SB was administrated 48 h post-transfection to induce the viral lytic cycle. (**b**) The translation and transcription of viral genes were assessed by immunoblotting (upper) and RT-PCR (lower), respectively. NA cells were transfected twice with siTSG101 or siGFP and once with TSG∆154-1054 prior to TPA/SB treatment.

In general, tumor viruses manipulate cellular functions for their own advantage. Accordingly, we examined the rationale of why EBV induced TSG∆154-1054 to stabilize TSG101. To address this question, we induced the late lytic cycle in NA cells by treatment with 12-O-tetradecanoylphorbol-13-acetate (TPA) and sodium butyrate (SB). The following immunoblot analysis showed that TSG∆154-1054-induced increase of the TSG101 protein was accompanied by upregulation of the EBV late structure proteins, VCA and gp350/220. This was comparable to the direct overexpression of TSG101 (Figure 4a). A translational mutant of TSG∆154-1054 (TSG∆154-1054-AAA) could not stabilize TSG101 or upregulate VCA and gp350/220. Neither were the early proteins, Zta, Rta and EA-D, affected (Figure 4a). This implies that the influence of TSG∆154-1054 on EBV gene expression is limited to late viral structural proteins.

To clarify whether TSG∆154-1054 or TSG101 is required for generating the viral late proteins, TSG101 was knocked down by siRNA (siTSG101) together with control siRNA (siGFP). Depletion of TSG101 protein led to downregulation of VCA and the disappearance of gp350/220, despite the presence of TSG∆154-1054 (Figure 4b). Notably, the transcription of the VCA and gp350/220 genes was also repressed when TSG101 was knocked down (Figure 4b). These data suggested that EBV induces TSG∆154-1054 to stabilize TSG101, and TSG101 *per se* is necessary for the transcription of viral late lytic genes leading to increased structural proteins.

### 2.5. TSG∆154-1054 Is Correlated with Increasing Anti-VCA Antibodies and EBV Load in NPC Patients

**Figure 5:**
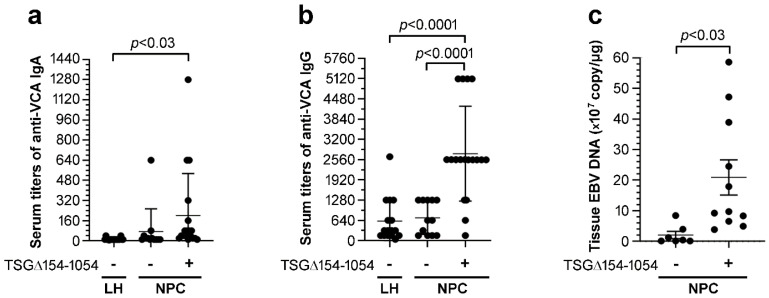
TSG∆154-1054 expression is positively associated with increasing anti-VCA antibody titers and EBV copy numbers in NPC patients. (**a**) Anti-VCA IgA titers were plotted against the absence or presence of TSG∆154-1054 in LH and NPC patients (means ± SD). (**b**) Anti-VCA IgG titers were plotted against the expression of TSG∆154-1054 in LH and NPC patients (means ± SD). (**c**) TSG∆154-1054 expression and EBV copy number in NPC tissues were plotted as means ± SEM. Serum titers of anti-VCA IgA and IgG were measured by immunoperoxidase assay. EBV copy number was assessed by real-time PCR targeted a viral EBNA-1 fragment, and the result was expressed as copy per μg of cellular DNA. The TSG∆154-1054 variant mRNA was detected by RT-nested-PCR ( undetectable, +: significant detection), and a nonparametric Mann Whitney U test was used to evaluate differences between groups.

To investigate the clinical significance of TSG∆154-1054 expression, the clinicopathological parameters of patients with NPC were compared after subdividing into TSG∆154-1054–positive (*n* = 18) and TSG∆154-1054–negative (*n* = 12) groups. Patients with lymphoid hyperplasia (LH) of the nasopharynx were enrolled as non-malignant controls (*n* = 18). LH tissues were generally TSG∆154-1054–negative and patients with LH usually had low or low to normal serum titers of anti-VCA IgA (≤20) and IgG (≤320) with few exceptions (Figure 5a,b). Anti-VCA IgA (>20) and anti-VCA IgG (>320) antibody titers can be used as screening markers to differentiate NPC patients from the healthy individuals for aiding in diagnosis of NPC [29,30]. Of note, most of the NPC patients expressing TSG∆154-1054 exhibited distinctively higher serum titers of anti-VCA IgA (≥40) and anti-VCA IgG (≥1280) as compared to those who were TSG∆154-1054-negative (*p* < 0.03, *p* < 0.0001; Figure 5a,b). In addition, a Spearman rank correlation analysis showed that TSG∆154-1054 expression was correlated with the titers of anti-VCA IgA (r = 0.5239, *p* < 0.0001) and anti-VCA IgG (r = 0.6358, *p* < 0.0001) in this study cohort.

Finally, NPC tissue expression of TSG∆154-1054 significantly correlated with a rise in EBV viral load (r = 0.7374, *p* < 0.0005 by Spearman rank correlation). The EBV copy number of TSG∆154-1054-positive tissues (2.1 × 10^8^ ± 17.33, means ± SEM) were approximately tenfold higher than those of TSG∆154-1054-negative tissues (2.0 × 10^7^ ± 4.46, means ± SEM, *p* < 0.03; Figure 5c). Overall, these data strongly suggest that the expression of TSG∆154-1054 has an advantage in EBV replication.

## 3. Discussion

The generation of aberrant TSG∆154-1054 mRNA is a typical feature of proliferative tumors but not of healthy normal cells [13,14,15,16,17,18]. In this study, we found that EBV controls the re-splicing mechanism of TSG101 mRNA to produce the TSG∆154-1054 isoform, which possesses carcinogenic properties [18,26]. As a result, on the one hand EBV utilizes the function of TSG∆154-1054 to stabilize TSG101 that in turn facilitates the transcription of viral late genes (VCA and gp350/220). On the other hand, TSG∆154-1054 may cause malignancy, since it increases full-length TSG101, which significantly promotes invasion and metastasis in cancer cells [18,26].

The protein product of TSG∆154-1054 lacks a leucine zipper near the C-terminus but retains a small part of the ubiquitin regulatory domain at the N-terminus [13,26]. This truncated small protein (3 kDa) is post-translationally modified in living cells to a large extent (~17 kDa) [26]. We previously demonstrated that the TSGΔ154-1054 protein competitively binds TSG101-associated E3 ligase (Tal), impeding the TSG101 interaction with Tal that inhibits the subsequent polyubiquitination and proteasomal degradation of TSG101 [26]. In addition, we showed that the stabilized TSG101 protein promotes cell proliferation, clonogenicity and tumor growth in nude mice, and these observations are further reflected in the cellular invasion, migration and metastasis of NPC patients [18,26].

The mechanism of TSGΔ154-1054-stabilizing TSG101 is not only beneficial to enhancing carcinogenesis [18,26], but also confers another advantage in the EBV life cycle. In this study, we demonstrate that production of infectious EBV virions is reinforced upon TSG101 stabilization caused by TSGΔ154-1054. To achieve this function, TSGΔ154-1054 has to be translated into protein, since the translational mutant TSGΔ154-1054-AAA fails to enrich the TSG101 protein level, leading to a blockage of EBV late gene expression. However, the low molecular mass and low expression level of TSGΔ154-1054 render this protein demanding to uncover the underlying molecular mechanism and its physiologic regulation. This will be a challenge in the therapeutic development of anti-tumor and anti-virus drugs targeting TSG101.

EBV exploits TSG101’s important role in transcriptional regulation to facilitate late gene transcription. TSG101 binds and stabilizes the glucocorticoid receptor to improve its DNA binding ability and transcriptional activity [31,32]. TSG101 is recruited by apoptosis-antagonizing transcription factor (AATF) to enhance mono-ubiquitination of the androgen receptor and activate androgen receptor-mediated transcription [33,34]. On the other hand, when TSG101 binds p300 and Daxx, it represses the transcriptional activities of the androgen receptor and glucocorticoid receptor, respectively [35,36]. It seems likely then that TSG101 regulates gene transcription positively or negatively depending on its interaction with transcriptional corepressors or coactivators. We previously reported that TSG101 binds Rta and that this assists in loading the Rta protein onto EBV late promoters for efficient late gene transcription [37]. This, then is the reason TSG101-depleted cells fail to express late viral proteins and infectious virus particles decrease in yield.

TSG101 was identified as a component of the endosomal sorting complex required for transport-I (ESCRT-I) which recognizes ubiquitinated cargo, and this is essential for the delivery and assembly of late viral proteins of RNA viruses, such as HIV and Ebola [38,39]. Notably, this endosomal sorting function of TSG101 is also critical for the effective infection of DNA tumor viruses, such as human papillomaviruses (HPV) [40] and Kaposi’s sarcoma-associated herpesvirus (KSHV) [41]. Instead of facilitating viral particle endocytosis into host cells, as seen for HPV and KSHV [40,41], TSG101 is employed by EBV for the release of mature viral particles [42,43]. The nuclear egress of EBV viral capsids is impaired when the ubiquitin binding ability of TSG101 is disrupted by prazoles, the proton pump inhibitors, which bind and induce structural change of TSG101 [42,43]. In fact, EBV utilizes TSG101 for two purposes during its productive lytic cycle: first for viral late gene transcription [37], and second for the delivery of viral late proteins [42,43]. Interestingly, these dual roles of TGS101 explicitly act on the EBV structural proteins, like VCA and gp350/220, which are encoded from the EBV open reading frames of the *BcLF1* and *BLLF1* genes, respectively. VCA is the major structural component of the nucleocapsid, whereas gp350/220 are *N*- and *O*-linked glycoproteins and also the most abundant components of the EBV envelope [1]. TSG101 plays an indispensable role in the processing of these viral late proteins, explaining why increasing TGS101 is broadly observable during EBV lytic reactivation no matter how it is induced; e.g., transfection of Zta and Rta in NPC cells or anti-IgG treatment in BL cells.

The clinical data of NPC patients further consolidates a role of TSG∆154-1054 in the expression of EBV late protein VCA. Patients expressing TSG∆154-1054 have high serum levels of anti-VCA IgG/IgA, indicating that high abundance of VCA antigen is recognized by the immune system, which is a sign of reactivation of chronic EBV infection. In addition, greater EBV load in TSG∆154-1054-expressing patients further validates the progression of viral lytic replication. Thus, the observation from cell cultures in this study can be recapitulated in vivo in humans.

To profit from the utilization of TSG101 in the viral life cycle, EBV triggers the production of TSG∆154-1054 via modulating alternative splicing. This is achieved through EBV viral proteins such as EBNA-1, Zta and Rta, although the detail mechanism is still unclear. We speculate that EBNA-1 could recruit splicing factors onto the re-splicing sites to generate TSG∆154-1054 mRNA. This is likely because EBNA-1 protein has been proven to alter the alternative splicing profiles of a number of cellular genes via physical interaction with splicing regulators such as hnRNP M, hnRNP H1, hnRNP U, hnRNP K, hnRNP C, DHX15, and SFPQ [4,27]. Mta (also known as SM), an EBV immediate early lytic protein, is another viral factor potentially affecting alternative splicing via interacting with splicing regulator SRSF3 (formerly SRp20) to modulate splice site selection. Through this pathway, Mta increases the alternatively spliced isoform STAT1β, but not the full-length STAT1α, which is the physiologically active form of STAT1 [44,45,46]. STAT1β lacks most of the transactivation domain of STAT1 and mice expressing STAT1β exhibit defects in IFNγ signaling and decreased NK cell-dependent anti-tumor activity [47]. Therefore, EBV increases the level of STAT1β, which may impair the interferon signaling network and counteract the host antiviral and antitumor responses. Since Mta may change the alternative splice site selection, it is worthwhile to examine the effect of Mta on the re-spliced sites of TSG101 mRNA. It is noteworthy that the levels of EBNA-1, Zta, Rta and Mta are all augmented during EBV lytic cycle progression [48], likely providing the opportunity to promote re-splicing on TSG101 mRNA to generate TSG∆154-1054.

In conclusion, we found that EBV supports re-splicing of TSG101 mRNA to produce the TSG∆154-1054 mRNA variant. TSG∆154-1054 is particularly augmented in response to the EBV lytic reactivation, and associated with EBV structural gene expression, which is essential for viral replication.

## 4. Materials and Methods

### 4.1. NPC Tissue Collection

Patients with primary NPC (*n* = 30) with stage II to IV, according to the American Joint Committee on Cancer (AJCC) staging system, were recruited in this study. Patients with nasopharyngeal LH (*n* = 18) who had been pathologically diagnosed as absence of tumor cells were enrolled as the control group. The fresh specimens of these patients were provided by the Departments of Otolaryngology and Pathology of National Taiwan University Hospital.

An immunoperoxidase assay of anti-VCA IgA and IgG titers was conducted with patients’ sera [29], which were collected at their first visit. This study was approved by the Institutional Review Board of the College of Medicine, National Taiwan University (approval number: 201311004RIND).

### 4.2. Cell Culture

NA and HA stable cell lines were established by co-cultivation of Akata-EBV (a gift from Prof. K. Takada) with NPC-TW01 and HONE-1 cells, respectively; so that the recombinant Akata-EBV strain infects these NPC cell lines [49,50]. Paired NP and HP stable cell lines, which are NPC-TW01 and HONE-1 cells carrying pZip-NeoSV(X)1 plasmid were used in this study as EBV-negative control [51]. Establishment of EBNA-1, Zta-2 and Zta-3 stable cell lines in RHEK-1 cells, and the SV40 immortalized keratinocytes, had been described previously [52,53]. All the NPC and RHEK-1 stable cell lines were cultured with Dulbecco’s modified Eagle’s medium (DMEM; Hyclone, Thermo Scientific, Waltham, MA, USA) supplied with L-glutamine, penicillin-streptomycin, and 10% fetal calf serum (Hyclone, Thermo Scientific, Waltham, MA, USA). Akata-pZip and Akata-EBV cells were cultured with RPMI 1640 medium (Hyclone, Thermo Scientific, Waltham, MA, USA) containing similar supplements as described above. All of these stable cell lines were maintained under G418-selection (Thermo Fisher Scientific, Waltham, MA, USA)

Akata is originally the EBV-producer cell, yet its EBV DNA was lost after serial passages [54]. The EBV-null Akata clone was selected for re-infection with recombinant EBV carrying a neomycin-resistant gene (designated Akata-EBV cell line) [49,50]. This cell line was also stably transfected with neomycin-contained pZip plasmid serving as EBV-negative control (Akata-pZip cell line) [50]. The lytic induction of Akata-EBV cells was achieved by adding 0.8% (vol/vol) rabbit anti-human IgG (Cappel, Aurora, OH, USA) to the culture medium to allow for the cross-linking of the IgG molecules on the surface of these cells. The lytic induction of NA cells was achieved by treatment with 12-*O*-tetradecanoylphorbol-13-acetate (TPA, 40 ng/mL, Sigma-Aldrich, St. Louis, MO, USA) plus sodium butyrate (SB, 3 mM, Sigma-Aldrich, St. Louis, MO, USA) for 48 h.

### 4.3. Construction and Transfection of Plasmids and siRNAs

Full-length TSG101 and truncated TSGΔ154-1054 fragments were amplified by PCR and subcloned into pcDNA3.1 vector (Invitrogen, Carlsbad, CA, USA). Plasmid constructs containing TSGΔ154-1054-AAA was subcloned by mutating the start codon ATG of TSGΔ154-1054 to AAA using QuikChange Lightening Site-Directed Mutagenesis kit (Agilent Technologies, Santa Clara, CA, USA). The EBNA-1 expression plasmid pCEP4 was purchased (Thermo Fisher Scientific, Waltham, MA, USA). Cloning of Zta and GFP-Rta expression plasmids, as well as siTSG101 and siGFP, are described in our previous publications [37,55]. Transfection of expression plasmids and siRNAs was carried out using TransFast Transfection Reagent (Promega, Madison, WI, USA). To successfully deplete the endogenous TSG101, siTSG101 together with siGFP were transfected twice at an interval of 24 h.

### 4.4. RT-Nested-PCR

Total RNAs were isolated with TRIzol reagent (Invitrogen, Carlsbad, CA, USA), followed by treatment with DNase I (Gibco BRL, Gaithersburg, MD, USA). To generate cDNAs, reverse transcription was performed with random hexamers (Gibco BRL, Gaithersburg, MD, USA) and SuperScript^TM^ II RNase H- reverse transcriptase (Gibco BRL, Gaithersburg, MD, USA), according to the manufacturer’s instructions. To detect alternative splicing events, TSG101-specific nested-PCR was performed as described in our previous work [18]. Primer sequences and PCR conditions are provided in Table 1.

### 4.5. RT-PCR Targeting EBV Viral Gene Products

The prepared cDNAs (described above) were used for detecting EBV viral transcripts. Each PCR amplification reaction was set up in a 25 μL mixture containing 1 μL cDNA, 1× PCR reaction buffer (Promega, Madison WI, USA), 0.2 mM of dNTPs (Promega, Madison WI, USA), 0.2 μM of forward and reverse primers, and 1 U of Prozyme DNA polymerase (Protech, Taipei, Taiwan). The primer sequences and PCR conditions for targeting Zta, VCA, gp350/220 and DAD-1 were described in Table 1.

### 4.6. Quantification of EBV Copy Number

Total DNA of NPC tissues was isolated using TRIzol reagent, according to the manufacturer’s instructions. EBNA-1 fragment was targeted in the following real-time quantitative PCR (qPCR) assay. The reaction set-up and data calculation of qPCR was operated as previously recommended [56]. Briefly, each reaction mixture containing 1 μL of cDNA, 0.2 μM of primers, 0.1 μM of dual-labeled fluorescence probe (Applied Biosystems, Foster City, CA, USA) and 2× TaqMan universal PCR master mixture (Applied Biosystems, Foster City, CA, USA) was prepared. Each sample was tested in duplicate. The thermal cycling parameters, as well as used primers and probes, are described in Table 1. To verify the reaction specificity and to exclude the possibility of DNA contamination, reaction without template was included as negative controls. DNA extracted from H2B4 cells [57], which contain one copy EBV/cell, was run in parallel as a standard calibration control. The amplification was performed in a GeneAmp PCR System 9700 (Applied Biosystems, Foster City, CA, USA).

### 4.7. Northern Blotting Analysis

Thirty μg of total RNAs were denatured at 65 °C for 15 min and subsequently separated in a 1% agarose gel containing 1x MOPS buffer (Thermo Fisher Scientific, Waltham, MA, USA) and 6% formaldehyde (Sigma-Aldrich, St. Louis, MO, USA). Capillary transfer of RNA from agarose gel to Immobilon-NY+ membrane (Millipore, Billerica, MA, USA) was performed with 20× SSC, and the complete process took at least 18 h. Following UV cross-linking, the blots were pre-hybridized at 42 °C for 2 h in ultrasensitive hybridization buffer ULTRAhyb (Ambion, Austin, TX, USA). Then, 1 × 10^7^ cpm of [^32^P]-labeled full-length TSG101 and TSG∆154-1054 probes were hybridized individually overnight to the blots in the ULTRAhyb at 42 °C. Finally, the blots were stringently washed in 0.1% SDS-containing 2× SSC for 10 min at 65 °C, four to six times, and the signals were visualized by autoradiography with X-ray films.

### 4.8. Immunoblotting Analysis

Cell lysates harvested by a RIPA lysis buffer were separated by 10% SDS-polyacrylamide gel electrophoresis. The electrophoretic transfer of proteins from SDS-polyacrylamide gel to nitrocellulose membranes (Hybond-C membrane, Amersham Biosciences, Piscataway, NJ, USA) was successively performed. The blots were blocked in washing buffer (100 mM Tris-HCl pH 7.4, 150 mM NaCl, 0.2% Tween-20) containing 4% skim milk at 37 °C for 30 min. Then, the blots were incubated at 4 °C overnight with primary antibodies against TSG101 (clone 4A10, 1:1000 dilution, GeneTex, Irvine, CA, USA), Rta (clone 467; 1:20 dilution) [58], Zta (clone 4F10; 1:20 dilution) [59], EA-D (clone 88; 1:20 dilution) [60], VCA (clone 343; without dilution) [60], gp350/220 (clone 201; without dilution) [60] and β-actin (1:10000 dilution; Sigma, St. Louis, MO, USA). After three rounds of 10 min washing with washing-buffer, the blots were incubated at room temperature for l h with the 1:10000 diluted horseradish peroxidase-conjugated secondary antibodies (Jackson ImmunoResearch, West Grove, PA, USA). Finally, the targeted proteins were detected by enhanced chemiluminescence (PerkinElmer, Waltham, MA, USA).

### 4.9. CHX Chase Assay to Assess TSG101 Stability

Cells were incubated with cycloheximide (300 μg/mL, Sigma-Aldrich, St. Louis, MO, USA) at 18 h post-transfection. Then, cell lysates were harvested at serial time points. The degradation of TSG101 protein was visualized by immunoblotting. To determine the half-life of TSG101, the blots were scanned by densitometry and analyzed after normalization to the corresponding β-actin levels.

### 4.10. Statistical Analysis

The statistical significances of the differences and correlation coefficients were calculated using GraphPad PRISM version 8.0.2 (GraphPad Software, Inc., San Diego, CA, USA). The statistical significance was set at *p* < 0.05. Densitometric analysis was achieved using Image J software (NIH Image, Bethesda, MD, USA).

## Figures and Tables

**Table 1:** PCR and qPCR conditions with primer/probe sequences.

Genes (Amplicon Sizes)	Primer Sequences	PCR Condition
**Nested-PCR** **TSG101** (Full-length: 1148 bp, TSG∆154-1054: 247 bp)	**First round PCR** Forward primer (P1): 5′-CGGTGTCGGAGAGCCAGCTCAAGAAA-3′ Reverse primer (P2): 5′-CCTCCAGCTGGTATCAGAGAAGTCAGT-3′ **Second round PCR** Forward primer (P3): 5′-AGCCAGCTCAAGAAAATGGTGTCCAAG-3′ Reverse primer (P4): 5′-TCACTGAGACCGGCAGTCTTTCTTGCTT-3′	95 °C, 50 s 65 °C, 30 s 72 °C, 1 min (25 cycles) 95 °C, 50 s 67 °C, 30 s 72 °C, 1 min (30 cycles)
**PCR**		
**VCA** (1268 bp)	Forward primer (LMRC 181): 5′-CGGGATCCGGTCGTGTACTTGGGATTG-3′ Reverse primer (LMRC 182): 5′-CGGGATCCCCCCATCTCCCTCTTACC-3′	95 °C, 1 min 57 °C, 1 min 72 °C, 1 min 30 s (30 cycles)
**gp350/220** (1008 bp)	Forward primer (LMRC 256): 5′-CGGGATCCGGCACTGAATAGGTAGCA-3′ Reverse primer (LMRC 257): 5′-CGGGATCCGAGACAATGGAGGCAG-3′	95 °C, 1 min 52 °C, 1 min 72 °C, 1 min 30 s (30 cycles)
**Zta** (182 bp)	Forward primer (Z1): 5′- TTCCACACAGCCTGCACCAGTG-3′ Reverse primer (Z2): 5′-GGCAGCAGCCACCTCACGGT-3′	95 °C, 1 min 55 °C, 1 min 72 °C, 1 min (30 cycles)
**DAD-1** (240 bp)	Forward primer: 5′-GCAGTTATGTCGGCGTCGGTAG-3′ Reverse primer: 5′-GTTCTGTGGGTTGATCTGTATTC-3′	94 °C, 15 s 60 °C, 15 s 72 °C, 30 s (25 cycles)
**qPCR**		
**EBNA-1**	Forward primer (EBNA-1162F): 5′-TCATCATCATCCGGGTCTCC-3′ Reverse primer (EBNA-1229R): 5′-CCTACAGGGTGGAAAAATGGC-3′ Probe (EBNA-1186T): 5′-(FAM)*CGCAGGCCCCCTCCAGGTAGAA(TAMRA)*-3′	95 °C, 15 s 60 °C, 1 min (40 cycles)

* FAM and TAMRA are the fluorescence dyes.
